# Is water‐soluble contrast enema examination for integrity of rectal anastomosis necessary prior to ileostomy reversal?

**DOI:** 10.1002/jgh3.12267

**Published:** 2019-11-06

**Authors:** Hui Lu Goh, Lauren Hawkins, Sivesh K Kamarajah, Sharad Karandikar, Mark Goldstein

**Affiliations:** ^1^ Heartlands Hospital University Hospitals Birmingham Foundation NHS Trust Birmingham UK; ^2^ Department of HPB and Transplant Surgery The Freeman Hospital Newcastle upon Tyne UK; ^3^ Institute of Cellular Medicine University of Newcastle Newcastle upon Tyne UK

**Keywords:** leaks, outcome, rectal cancer, water‐soluble contrast

## Abstract

**Background and Aim:**

Routine use of water‐soluble contrast enema (WSCE) to assess anastomotic integrity is debated. This study aimed to evaluate the role of WSCE to assess anastomotic integrity following anterior resections (AR) with defunctioning stoma prior to reversal and identify factors to limit its selective use.

**Methods:**

This retrospective study evaluated all WSCE performed over a 7‐year period at a high‐volume colorectal unit. Risk factors for radiological abnormality/leak, including malignancy, chemoradiotherapy, and immediate postoperative complications, were recorded. A gastrointestinal specialist radiologist and surgeon validated all WSCEs reported as abnormal.

**Results:**

Of the 486 WSCE studies identified, 92 were excluded (repeat studies (*n* = 51), pediatric cases [*n* = 2], no AR [*n* = 39]). A total of 394 WSCE studies were evaluated (260 cancer; 134 noncancer patients); 14% (37/260) of cancer patients and 8% (10/134) of noncancer patients had abnormal studies (*P* = 0.072). Of the 37 abnormal studies in cancer patients, 73% (27/37) radiological leaks were found, and 41% (*n* = 11/27) of these patients had postoperative complications. Of the 10 abnormal studies in noncancer patients, 20% (2/10) radiological leaks were found, but none of these patients had postoperative complications. Overall leak rates were 7% (29/394), and rates were significantly higher in cancer patients than noncancer patients (10 *vs* 2%, *P* = 0.005).

**Conclusion:**

Routine use of WSCE may not be necessary prior to reversal. WSCE should be selectively used in event of postoperative leak or complications. Noncancer resections are less likely demonstrate a leak.

## Graphical abstract

Routine use of water‐soluble contrast enema (WSCE) may not be necessary prior to reversal. WSCEs should be selectively used in the event of a postoperative leak or complications. Noncancer resections are less likely to demonstrate a leak.

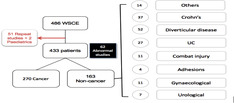

## Introduction

Anastomotic leaks (AL) following colorectal resection pose demonstrate short‐ and long‐term sequelae and increased postoperative morbidity and mortality.^1^, ^2^ The risk factors for developing an anastomotic leak are multifactorial, and it is difficult to identify a single causative risk factor for anastomotic leaks.^1^ A proximal defunctioning stoma can reduce the risk of serious sequelae, such as pelvic sepsis and collections. Stoma formations do not reduce the rate of anastomosis dehiscence postresection but can reduce it to subclinical anastomotic leaks.^3^ Traditionally, anastomotic integrity has been assessed endoscopically or radiologically to prevent complications from the primary anastomosis prior to reversal.

Water‐soluble contrast enemas (WSCEs) are a frequently used investigation method to assess anastomotic integrity and have been shown to be superior to computed tomography imaging for distal anastomoses.^4^ In recent years, there has been much debate regarding the routine use of WSCEs to assess anastomotic integrity compared to a simple digital rectal examination.^5^, ^6^, ^7^ However, these studies have focused predominantly on cases following resection for colorectal cancer, with limited data in noncancer resections.

The primary aim of this study is to examine the role of WSCEs to assess integrity following colorectal anterior resection (AR) in both cancer and noncancer patients. The secondary aim was to identify patient groups to be able to use WSCEs selectively based on their demographic, pathology, and postoperative complications.

## Methods

This retrospective study evaluated WSCEs over a 7‐year period (October 2009 to April 2016) performed in a multisite, high‐volume colorectal unit. Consecutive patients undergoing WSCE were identified from the radiology database. Clinical notes and electronic records were also used to collate the inpatient data. Demographic, clinical, operative, and postoperative data were recorded and evaluated to identify risk factors for a radiological abnormality and/or leak, including malignancy, chemoradiotherapy, and postoperative complications. Postoperative complications were defined as the presence of complications according to the Clavien‐Dindo Classification grade.^8^ A radiologist with gastrointestinal specialization and a colorectal surgeon with more than 15 years of experience reviewed and validated all WSCEs reported as abnormal. Pediatric patients, repeat studies on the same patient, and patients who did not have anastomosis following AR were excluded. All WSCEs were performed by one of four consultant gastrointestinal radiologists with rectal intubation using a Foley catheter of varying sizes, with dilute gastrografin passed rectally.

### *Statistical analysis*

Continuous variables were expressed as mean ± SD or median (interquartile range) and analyzed using *t*‐test or Mann–Whitney test where appropriate. Categorical variables were expressed as percentages and analyzed using chi‐square test or Fisher's exact test where appropriate. For all analyses, a *P*‐value <0.05 was considered statistically significant. Data analysis was undertaken using R Foundation Statistical Software (R 3.2.1, R Foundation for Statistical Computing, Vienna, Austria) as previously described.^9^, ^10^, ^11^, ^12^, ^13^, ^14^, ^15^, ^16^, ^17^, ^18^, ^19^, ^20^

## Results

Of the WSCE studies performed, 81% (394/486) of WSCEs were included in the study cohort. Excluded cases were 51 repeat studies, two pediatric cases, and 39 cases that did not have an AR. The median age of the entire cohort was 60 years (interquartile range: 50–70 years), and a majority of patients were male (61%, 241/394). This study broadly classifies patients into two main groups: cancer (*n* = 260) *versus* non‐cancer resections (*n* = 134) (Table 1). Cancer patients were significantly older than noncancer patients (66 *vs* 46 years old, *P* < 0.001).

**Table 1:** Indications of water‐soluble contrast enema

Indications	Cancer, *n* = 270 (%)	Noncancer, *n* = 163	*P*‐value
Check anastomosis integrity	260 (96)	134 (82)	<0.001‡
Others†	10 (4)	29 (18)	

† Others include strictures, fistula, hernia, and inflammatory bowel disease.

‡ Statistical test used was chi‐square test.

**Figure 1:**
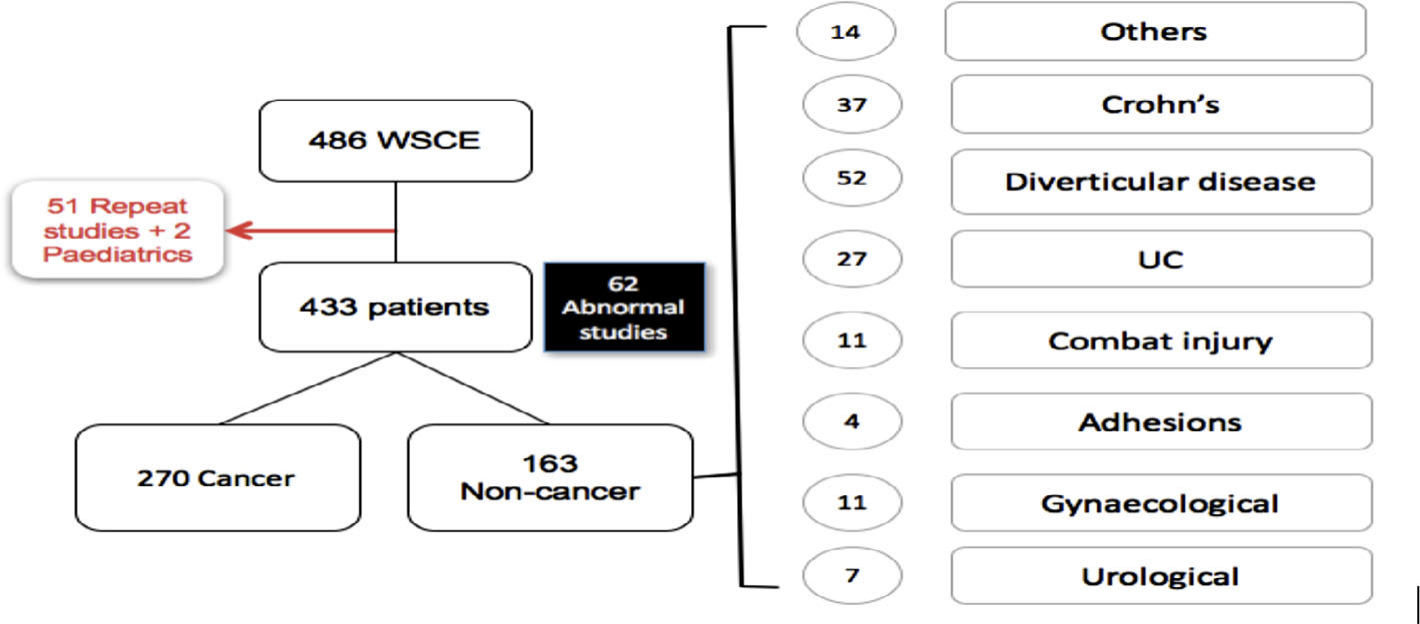
Results by disease. UC, ulcerative colitis; WSCE, water‐soluble contrast enema.

There was no significant difference in gender between both groups. As seen in Figure 1, patients in the noncancer category had bowel resections following diverticular disease (strictures), inflammatory bowel disease (ulcerative colitis and Crohn's disease), colonic perforation, adhesions, fistula, incarcerated hernia, gynecological complications (resection following endometriosis stricture), urological complications (colonic injury during bladder or prostate surgery), and miscellaneous indications.

Of the WSCE studies performed, 12% (*n* = 47/394) were abnormal. There were no significant differences in rates of abnormal studies between cancer and noncancer patients (14 *vs* 8%, *P* = 0.072) (Table 2). Of the 37 abnormal studies on cancer patients, 73% (27/37) were radiological leaks compared to 10 abnormal studies in non‐cancer patients, of which 20% (2/10) were radiological leaks.

**Table 2:** Abnormal studies in cancer and noncancer group stratified by indications

Cancer group					
	Radiological leak, *n* = 28 (%)	Stricture, *n* = 9 (%)	Fistula, *n* = 3 (%)	Others† *n* = 3 (%)	*P*‐value
Check anastomosis integrity	27 (96)	7 (78)	2 (67)	1 (33)	0.012‡
Specific pathology	1 (4)	2 (12)	1 (33)	2 (67)	

Noncancer group					
	Radiological leak, *n* = 2 (%)	Stricture, *n* = 6 (%)	Fistula, *n* = 5 (%)	Others† *n* = 6 (%)	*P*‐value
Check anastomosis integrity	2 (100)	3 (50)	1 (20)	4 (67)	0.219‡
Specific pathology	0 (0)	3 (50)	4 (80)	2 (33)	

† Others include hernia and inflammatory bowel disease.

‡ Statistical test used was chi‐square test.

Of the WSCE studies conducted, only 7% (*n* = 29/394) had a leak radiologically (Table 3). The rates of radiological leaks were significantly higher in cancer patients than noncancer patients (10 *vs* 2%, *P* = 0.005). In patients with a radiological leak, there were no significant differences in age between cancer and noncancer patients (median: 65 *vs* 56 years old, *P* = 0.2). In the cancer patients, 41% (*n* = 11/27) with abnormal studies had postoperative complications or an identified leak postsurgery. All noncancer patients with abnormal studies had no postoperative complications or an identified leak postsurgery. In noncancer patients, 7% (*n* = 8/124) with normal studies had postoperative complications. Similarly, 9% (*n* = 19/223) of cancer patients with normal studies had postoperative complications.

**Table 3:** Associated postoperative complications in those with abnormal and normal water‐soluble contrast enema studies

Abnormal studies—radiological leak	Cancer, *n* = 27 (%)	Other patients, *n* = 2 (%)	*P*‐value
Inpatient complication	12 (44)	0 (0)	0.654
No complication	15 (56)	2 (100)	

Normal studies	Cancer, *n* = 223 (%)	Other patients, *n* = 124 (%)	*P*‐value
Inpatient complication	23 (10)	8 (7)	0.072
No complication	200 (90)	116 (93)	

## Discussion

Reversal of a defunctioning loop ileostomy in the presence of a radiological leak is associated with poor surgical outcome, increased health care, and financial costs.^21^ In low AR for rectal cancer, a temporary defunctioning ileostomy is recommended.^22^ The septic consequences of anastomotic dehiscence can be reduced by performing a loop ileostomy to divert fecal stream.^23^ Despite an increasing trend of diverting stomas being fashioned, no clear guidance exists as to when and how to assess anastomotic integrity and patency and the best way to manage patient with an established leak.

A WSCE is commonly performed prior to the reversal of defunctioning stoma for assessment of anastomotic integrity and to rule out any leak, blockage, or fistula formation.^23^, ^24^ These examinations are sometimes difficult to interpret in the presence of a pouch or when there is a “dog ear” from a colorectal anastomosis using the double‐stapling technique. This is usually performed 6–8 weeks after the primary operation, prior to ileostomy closure.^25^ It has been demonstrated that the use of WSCE in the immediate postoperative period has a low predictive value in detecting subclinical leaks and might disrupt an intact anastomosis by the pressure produced when installing the contrast.^26^ Furthermore, having a WSCE performed in the early postoperative period with an underlying sepsis could potentially spread infection hematogenously from excessive air insufflation.^27^

Its routine use, however, is debated. Several studies have shown that WSCE does not add value or alter patient management when the results of endoscopic or digital rectal examination are normal.^25^, ^27^, ^28^ In other studies, however, a contrast enema was effective in excluding clinically significant anastomotic problems, especially after clinical anastomotic leaks.^5^

The overall radiological leak rate on WSCE in this study was 7% (29/394). All leaks were identified by clinical acumen and confirmed radiographically. If a leak is suspected, a pelvic computed tomography (CT) scan is often used initially. A CT scan can not only detect a leak but can also accurately outline the presence and extent of pelvic abscess.^28^ In this study, seven patients in the cancer group had a CT scan performed postoperatively, which confirmed anastomotic leak. In four of these patients, subsequent contrast enemas were abnormal—three showed an anastomotic leak and one a fistula.

The majority of patients with a proven radiological leak on WSCE performed prior to reversal of ileostomy were male (80%, 24/30), with only 20% (6/30) female. Our findings agree with a systematic review conducted by Pommergaard et al. that male gender is one of the preoperative risk factors for anastomotic leakage after resection for colorectal cancer.^29^ It has been inferred that male gender poses a higher risk to anastomotic leakage due to the deeper and narrow pelvic anatomy, contributing to technical difficulties.^30^, ^31^ This study also found that 93% (27/29) with a confirmed radiological leak on WSCE were cancer resections, whereas 7% (2/29) were noncancer resections.

Several studies have evaluated a variety of risk factors of anastomotic leaks (AL); however, there is no universal agreement regarding the associated risk factors. Although it is generally accepted that variables such as low anastomotic level, smoking status, and presence of comorbidities are associated with leakage,^32^, ^33^, ^34^ other risk factors, including male gender and neoadjuvant chemoradiotherapy (nCRT), are not widely recognized.^35^ Although many studies have investigated risk factors of AL, it remains a life‐threatening complication that can arise in patients with no known risk factors.

Anastomotic leakage following AR has been reported with increased rates after nCRT.^36^, ^37^ It has been explained that preoperative radiotherapy results in local inflammation and tissue fibrosis, and could reduce wound healing, thus increasing the risk of anastomotic leakage.^37^ We identified five patients in the cancer group who received preoperative chemoradiotherapy prior to primary resection. Three of these patients who had preoperative chemoradiotherapy had an uneventful postoperative course and a subsequent normal WSCE study. One patient who received radiotherapy developed a postoperative complication or clinical leak following primary surgery but had a subsequent normal WSCE study. The second patient, however, developed a postoperative complication, with a confirmed leak on subsequent WSCE. Our study suggests that no evaluation by WSCE may be needed in those receiving neoadjuvant chemotherapy if there was no postoperative complication; however, further evaluation of this is required given the limited number of cases.

In addition to anastomotic defects, it is also essential to evaluate colorectal anastomosis for radiological stricture (not functional). WSCE detected seven strictures in the cancer group and three strictures in the noncancer group. This comprises of 3% (15/433) of all WSCE studies. This is comparable to a cross‐sectional review performed across 11 studies, in which the rate of detected strictures varied from 0 to 25%.^5^ Our WSCE studies also identified two fistulas: one with abnormal anatomy in the cancer group and one fistula and four other strictures (adhesions, abnormal anatomy and mass) in noncancer patients.

The limitation of the study is that, being retrospective, it is unable to evaluate variation in surgical technique and experience of the surgeon, chemoradiotherapy regimen, and radiological technique for performing the WSCEs. The extent and variability of the pelvic pathology and preoperative risk factors for anastomotic dehiscence smoking or BMI could not be studied. Finally, multivariable analysis was not possible due to an insufficient number of cases to adjust for other confounding factors. Hence it is not clear if cancer surgery is associated with a higher likelihood of requiring WSCE.

In conclusion, this study appears to demonstrate that WSCE prior to reversal to demonstrate an anastomotic leak in the absence of a clinical postoperative anastomotic leak may not necessary. Patients with postoperative complications or at a high risk of anastomotic dehiscence are best served by WSCEs prior to reversal. Selective use of WSCEs will reduce patient radiation exposure and the cost to the health‐care system. Future studies should aim to further validate our findings in larger cohorts.
